# Bis(μ_2_-3,5-diisopropyl-4*H*-1,2,4-triazole-κ^2^ *N* ^1^:*N* ^2^)bis­[(nitrato-κ*O*)silver(I)]

**DOI:** 10.1107/S1600536809028384

**Published:** 2009-07-22

**Authors:** Zhao-Yang Wang, Ying-Li Wang, Guang Yang, Seik Weng Ng

**Affiliations:** a Department of Chemistry, Zhengzhou University, Zhengzhou 450001, People’s Republic of China; b Department of Chemistry, University of Malaya, 50603 Kuala Lumpur, Malaysia

## Abstract

The neutral *N*-heterocycle in the title centrosymmetric dinuclear compound, [Ag_2_(NO_3_)_2_(C_8_H_15_N_3_)_2_], bridges two metal atoms through its imino N atoms. The N—Ag—N skeleton is bent [N—Ag—N = 127.2 (3)°]; as one of two O atoms of the nitrate anion is nearly coplanar with this N—Ag—N skeleton [Ag—O = 2.63 (1) Å], the coordination geometry around the Ag^I^ atom is regarded as trigonal-planar. One of the two isopropyl groups is disordered over two positions in respect of the methyl groups in a 1:1 ratio. In the crystal structure, inter­molecular N—H⋯O hydrogen bonding is observed between the nitrate groups and triazole ligands.

## Related literature

For the background to such silver–triazole compounds, see: Yang *et al.* (2007 ▶).
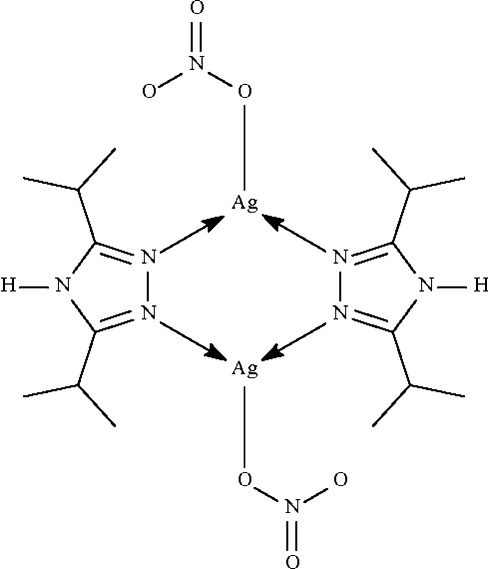


## Experimental

### 

#### Crystal data

[Ag_2_(NO_3_)_2_(C_8_H_15_N_3_)_2_]
*M* *_r_* = 646.22
Monoclinic, 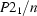

*a* = 5.791 (1) Å
*b* = 14.541 (1) Å
*c* = 14.578 (1) Å
β = 99.523 (2)°
*V* = 1210.6 (2) Å^3^
*Z* = 2
Mo *K*α radiation
μ = 1.66 mm^−1^
*T* = 293 K
0.41 × 0.17 × 0.13 mm

#### Data collection

Bruker SMART diffractometer
Absorption correction: multi-scan (*SADABS*; Sheldrick, 1996 ▶) *T*_min_ = 0.670, *T*_max_ = 1.000 (expected range = 0.540–0.805)
5562 measured reflections
2124 independent reflections
1389 reflections with *I* > 2σ(*I*)
*R*_int_ = 0.063

#### Refinement

*R*[*F* ^2^ > 2σ(*F* ^2^)] = 0.078
*wR*(*F* ^2^) = 0.240
*S* = 1.08
2124 reflections
151 parameters
18 restraints
H-atom parameters constrained
Δρ_max_ = 0.91 e Å^−3^
Δρ_min_ = −0.96 e Å^−3^

### 

Data collection: *APEX2* (Bruker, 1999 ▶); cell refinement: *SAINT* (Bruker, 1999 ▶); data reduction: *SAINT*; program(s) used to solve structure: *SHELXS97* (Sheldrick, 2008 ▶); program(s) used to refine structure: *SHELXL97* (Sheldrick, 2008 ▶); molecular graphics: *X-SEED* (Barbour, 2001 ▶); software used to prepare material for publication: *publCIF* (Westrip, 2009 ▶).

## Supplementary Material

Crystal structure: contains datablocks I, global. DOI: 10.1107/S1600536809028384/xu2560sup1.cif

Structure factors: contains datablocks I. DOI: 10.1107/S1600536809028384/xu2560Isup2.hkl

Additional supplementary materials:  crystallographic information; 3D view; checkCIF report

## Figures and Tables

**Table 1:** Hydrogen-bond geometry (Å, °)

*D*—H⋯*A*	*D*—H	H⋯*A*	*D*⋯*A*	*D*—H⋯*A*
N3—H3⋯O1^i^	0.89	2.06	2.93 (1)	167

Symmetry code: (i) 
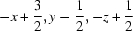
.

**Fig. 1.**
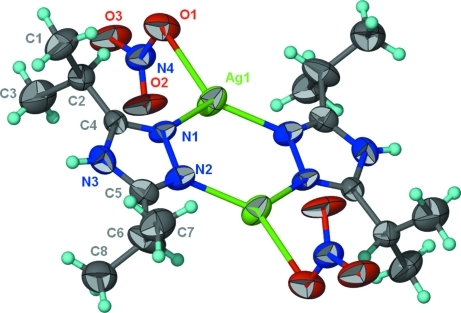
Thermal ellipsoid plot of [Ag(C8H15N3)(NO3)]2; ellipsoids are drawn at the 50% probability level. The disorder is not shown.

## supplementary crystallographic information

### Experimental

An acetonitrile solution (2 ml) of 3,5-diisopropyl-1*H*-1,2,4-triazole (0.1 mmol, 15 mg) was mixed with a acetoninitrile solution (1 ml) of silver nitrate (0.1 mmol, 17 mg). Ether was allowed to diffuse into the resulting solution. Colorless crystals were formed after a week in 50% yield. Calc. for C_16_H_30_N_8_Ag_2_O_6_: C 29.7; H 4.6, N, 17.3%. Found: C 29.7, H 4.7, N, 17.6%.

### Refinement

The H atoms were placed in calculated positions [C—H 0.96–0.98 Å; *U*(H) = 1.2–1.5*U*_eq_(C)]. The amino H-atom was similarly treated [N–H 0.89 Å].

One of the two isopropyl groups is disordered over two positions in the methyl groups only; the disorder was assumed to be 1:1. The C–C distances were restrained to 1.54±0.01 Å, and the 1,3-related C···C distances to 2.51±0.01 Å. The temperature factors of the primed atoms were restrained to those of the unprimed ones; the anisotropic temperature factors were restrained to be nearly isotropic.

### Crystal data

**Table:**

[Ag_2_(NO_3_)_2_(C_8_H_15_N_3_)_2_]	*F*(000) = 648
*M**_r_* = 646.22	*D*_x_ = 1.773 Mg m^−^^3^
Monoclinic, *P*2_1_/*n*	Mo *K*α radiation, λ = 0.71073 Å
Hall symbol: -P 2yn	Cell parameters from 1859 reflections
*a* = 5.791 (1) Å	θ = 2.8–21.8°
*b* = 14.541 (1) Å	µ = 1.66 mm^−^^1^
*c* = 14.578 (1) Å	*T* = 293 K
β = 99.523 (2)°	Prism, colorless
*V* = 1210.6 (2) Å^3^	0.41 × 0.17 × 0.13 mm
*Z* = 2	

### Data collection

**Table:**

Bruker SMART diffractometer	2124 independent reflections
Radiation source: fine-focus sealed tube	1389 reflections with *I* > 2σ(*I*)
graphite	*R*_int_ = 0.063
φ and ω scans	θ_max_ = 25.0°, θ_min_ = 2.0°
Absorption correction: multi-scan (*SADABS*; Sheldrick, 1996)	*h* = −6→6
*T*_min_ = 0.670, *T*_max_ = 1.000	*k* = −9→17
5562 measured reflections	*l* = −17→13

### Refinement

**Table:**

Refinement on *F*^2^	Primary atom site location: structure-invariant direct methods
Least-squares matrix: full	Secondary atom site location: difference Fourier map
*R*[*F*^2^ > 2σ(*F*^2^)] = 0.078	Hydrogen site location: inferred from neighbouring sites
*wR*(*F*^2^) = 0.240	H-atom parameters constrained
*S* = 1.08	*w* = 1/[σ^2^(*F*_o_^2^) + (0.1131*P*)^2^ + 8.3674*P*] where *P* = (*F*_o_^2^ + 2*F*_c_^2^)/3
2124 reflections	(Δ/σ)_max_ = 0.001
151 parameters	Δρ_max_ = 0.91 e Å^−^^3^
18 restraints	Δρ_min_ = −0.96 e Å^−^^3^

### Fractional atomic coordinates and isotropic or equivalent isotropic displacement parameters (Å^2^)

**Table:**

	*x*	*y*	*z*	*U*_iso_*/*U*_eq_	Occ. (<1)
Ag1	0.6205 (2)	0.57863 (7)	0.43648 (7)	0.0682 (5)	
O1	0.8666 (17)	0.6779 (7)	0.3367 (8)	0.080 (3)	
O2	1.0702 (19)	0.5875 (8)	0.4336 (8)	0.094 (4)	
O3	1.2426 (18)	0.6792 (9)	0.3497 (8)	0.096 (4)	
N1	0.5362 (17)	0.4427 (6)	0.3705 (6)	0.047 (2)	
N2	0.4403 (17)	0.3760 (7)	0.4232 (7)	0.054 (3)	
N3	0.5156 (19)	0.3113 (7)	0.2993 (8)	0.061 (3)	
H3	0.5270	0.2681	0.2570	0.074*	
N4	1.061 (2)	0.6493 (8)	0.3760 (8)	0.067 (3)	
C1	0.547 (3)	0.4325 (13)	0.1257 (10)	0.087 (5)	
H1A	0.3891	0.4523	0.1263	0.130*	
H1B	0.5473	0.3686	0.1093	0.130*	
H1C	0.6133	0.4680	0.0809	0.130*	
C2	0.692 (2)	0.4463 (9)	0.2217 (9)	0.058 (3)	
H2	0.6933	0.5126	0.2339	0.070*	
C3	0.946 (3)	0.4169 (15)	0.2284 (14)	0.104 (6)	
H3A	1.0273	0.4274	0.2904	0.155*	
H3B	1.0189	0.4520	0.1852	0.155*	
H3C	0.9529	0.3527	0.2137	0.155*	
C4	0.5824 (19)	0.4017 (7)	0.2966 (8)	0.044 (3)	
C5	0.429 (2)	0.3010 (8)	0.3788 (9)	0.055 (3)	
C6	0.342 (2)	0.2139 (8)	0.4109 (12)	0.085 (5)	
H6	0.3262	0.2233	0.4761	0.102*	0.50
H6'	0.3460	0.1810	0.3525	0.102*	0.50
C7	0.097 (3)	0.192 (2)	0.359 (2)	0.087 (8)	0.50
H7A	0.0448	0.1347	0.3805	0.131*	0.50
H7B	0.1006	0.1882	0.2934	0.131*	0.50
H7C	−0.0092	0.2401	0.3700	0.131*	0.50
C8	0.503 (4)	0.1308 (18)	0.409 (3)	0.089 (7)	0.50
H8A	0.4306	0.0774	0.4308	0.134*	0.50
H8B	0.6496	0.1423	0.4490	0.134*	0.50
H8C	0.5300	0.1205	0.3468	0.134*	0.50
C7'	0.083 (2)	0.203 (2)	0.408 (2)	0.087 (8)	0.50
H7'1	0.0510	0.1425	0.4297	0.131*	0.50
H7'2	0.0038	0.2097	0.3446	0.131*	0.50
H7'3	0.0273	0.2484	0.4461	0.131*	0.50
C8'	0.490 (4)	0.144 (2)	0.471 (2)	0.089 (7)	0.50
H8'1	0.3938	0.0928	0.4820	0.134*	0.50
H8'2	0.5548	0.1720	0.5292	0.134*	0.50
H8'3	0.6138	0.1236	0.4399	0.134*	0.50

### Atomic displacement parameters (Å^2^)

**Table:**

	*U*^11^	*U*^22^	*U*^33^	*U*^12^	*U*^13^	*U*^23^
Ag1	0.0868 (9)	0.0610 (7)	0.0623 (7)	−0.0294 (6)	0.0285 (5)	−0.0052 (5)
O1	0.051 (6)	0.080 (7)	0.107 (8)	0.001 (5)	0.007 (5)	0.031 (6)
O2	0.078 (7)	0.108 (9)	0.091 (7)	−0.021 (6)	0.003 (6)	0.066 (7)
O3	0.064 (6)	0.119 (9)	0.104 (8)	−0.015 (6)	0.016 (6)	0.057 (7)
N1	0.063 (6)	0.039 (5)	0.039 (5)	−0.016 (4)	0.009 (4)	0.003 (4)
N2	0.054 (6)	0.052 (6)	0.055 (6)	−0.018 (5)	0.009 (5)	0.007 (5)
N3	0.066 (7)	0.050 (6)	0.067 (7)	0.004 (5)	0.008 (6)	−0.009 (5)
N4	0.056 (7)	0.075 (8)	0.070 (7)	−0.001 (6)	0.016 (6)	0.018 (6)
C1	0.083 (10)	0.123 (14)	0.055 (8)	−0.004 (10)	0.013 (7)	0.012 (9)
C2	0.059 (8)	0.059 (8)	0.055 (7)	0.004 (6)	0.008 (6)	0.001 (6)
C3	0.056 (9)	0.146 (18)	0.112 (14)	0.007 (10)	0.027 (9)	0.040 (13)
C4	0.038 (6)	0.044 (6)	0.048 (6)	0.002 (4)	0.004 (5)	−0.001 (5)
C5	0.050 (7)	0.043 (7)	0.071 (8)	−0.010 (5)	0.005 (6)	0.001 (6)
C6	0.077 (10)	0.044 (7)	0.131 (14)	−0.003 (7)	0.011 (9)	0.021 (9)
C7	0.080 (9)	0.093 (10)	0.090 (12)	−0.017 (8)	0.020 (8)	0.011 (9)
C8	0.083 (10)	0.087 (10)	0.098 (12)	0.002 (8)	0.016 (9)	0.014 (9)
C7'	0.080 (9)	0.093 (10)	0.090 (12)	−0.017 (8)	0.020 (8)	0.011 (9)
C8'	0.083 (10)	0.087 (10)	0.098 (12)	0.002 (8)	0.016 (9)	0.014 (9)

### Geometric parameters (Å, °)

**Table:**

Ag1—N1	2.218 (9)	C3—H3B	0.9600
Ag1—N2^i^	2.232 (10)	C3—H3C	0.9600
Ag1—O2	2.615 (11)	C5—C6	1.469 (16)
Ag1—O1	2.630 (10)	C6—C7'	1.505 (10)
O1—N4	1.245 (14)	C6—C8'	1.508 (10)
O2—N4	1.224 (14)	C6—C7	1.529 (10)
O3—N4	1.258 (14)	C6—C8	1.529 (10)
N1—C4	1.297 (14)	C6—H6	0.9800
N1—N2	1.407 (12)	C6—H6'	0.9800
N2—C5	1.264 (15)	C7—H7A	0.9600
N2—Ag1^i^	2.232 (10)	C7—H7B	0.9600
N3—C5	1.342 (17)	C7—H7C	0.9600
N3—C4	1.373 (15)	C8—H8A	0.9600
N3—H3	0.8900	C8—H8B	0.9600
C1—C2	1.520 (19)	C8—H8C	0.9600
C1—H1A	0.9600	C7'—H7'1	0.9600
C1—H1B	0.9600	C7'—H7'2	0.9600
C1—H1C	0.9600	C7'—H7'3	0.9600
C2—C4	1.498 (18)	C8'—H8'1	0.9600
C2—C3	1.52 (2)	C8'—H8'2	0.9600
C2—H2	0.9800	C8'—H8'3	0.9600
C3—H3A	0.9600		
			
N1—Ag1—N2^i^	127.2 (3)	N1—C4—C2	125.2 (10)
N1—Ag1—O2	100.7 (4)	N3—C4—C2	126.2 (11)
N2^i^—Ag1—O2	108.0 (4)	N2—C5—N3	110.6 (11)
N1—Ag1—O1	110.5 (4)	N2—C5—C6	124.8 (13)
N2^i^—Ag1—O1	121.8 (4)	N3—C5—C6	124.6 (13)
O2—Ag1—O1	48.0 (3)	C5—C6—C7'	118.6 (15)
N4—O1—Ag1	95.3 (7)	C5—C6—C8'	124.9 (15)
N4—O2—Ag1	96.6 (8)	C7'—C6—C8'	114.3 (10)
C4—N1—N2	106.9 (9)	C5—C6—C7	111.1 (17)
C4—N1—Ag1	135.2 (7)	C5—C6—C8	115.7 (17)
N2—N1—Ag1	117.1 (7)	C7'—C6—C8	121 (2)
C5—N2—N1	107.8 (10)	C7—C6—C8	110.4 (10)
C5—N2—Ag1^i^	136.3 (9)	C5—C6—H6	106.3
N1—N2—Ag1^i^	115.6 (7)	C7—C6—H6	106.3
C5—N3—C4	106.1 (10)	C8—C6—H6	106.3
C5—N3—H3	126.9	C6—C7—H7A	109.5
C4—N3—H3	126.9	C6—C7—H7B	109.5
O2—N4—O1	119.8 (11)	H7A—C7—H7B	109.5
O2—N4—O3	121.1 (12)	C6—C7—H7C	109.5
O1—N4—O3	118.8 (11)	H7A—C7—H7C	109.5
C2—C1—H1A	109.5	H7B—C7—H7C	109.5
C2—C1—H1B	109.5	C6—C8—H8A	109.5
H1A—C1—H1B	109.5	C6—C8—H8B	109.5
C2—C1—H1C	109.5	H8A—C8—H8B	109.5
H1A—C1—H1C	109.5	C6—C8—H8C	109.5
H1B—C1—H1C	109.5	H8A—C8—H8C	109.5
C4—C2—C1	112.3 (11)	H8B—C8—H8C	109.5
C4—C2—C3	110.7 (11)	C6—C7'—H7'1	109.5
C1—C2—C3	113.7 (13)	C6—C7'—H7'2	109.5
C4—C2—H2	106.6	H7'1—C7'—H7'2	109.5
C1—C2—H2	106.6	C6—C7'—H7'3	109.5
C3—C2—H2	106.6	H7'1—C7'—H7'3	109.5
C2—C3—H3A	109.5	H7'2—C7'—H7'3	109.5
C2—C3—H3B	109.5	C6—C8'—H8'1	109.5
H3A—C3—H3B	109.5	C6—C8'—H8'2	109.5
C2—C3—H3C	109.5	H8'1—C8'—H8'2	109.5
H3A—C3—H3C	109.5	C6—C8'—H8'3	109.5
H3B—C3—H3C	109.5	H8'1—C8'—H8'3	109.5
N1—C4—N3	108.5 (10)	H8'2—C8'—H8'3	109.5
			
N1—Ag1—O1—N4	−89.2 (9)	N2—N1—C4—C2	−178.6 (10)
N2^i^—Ag1—O1—N4	82.9 (9)	Ag1—N1—C4—C2	−9.6 (18)
O2—Ag1—O1—N4	−3.1 (8)	C5—N3—C4—N1	−0.4 (13)
N1—Ag1—O2—N4	111.2 (9)	C5—N3—C4—C2	179.2 (11)
N2^i^—Ag1—O2—N4	−113.7 (9)	C1—C2—C4—N1	−126.3 (14)
O1—Ag1—O2—N4	3.2 (8)	C3—C2—C4—N1	105.4 (15)
N2^i^—Ag1—N1—C4	−170.4 (10)	C1—C2—C4—N3	54.0 (16)
O2—Ag1—N1—C4	−47.8 (11)	C3—C2—C4—N3	−74.2 (17)
O1—Ag1—N1—C4	1.2 (12)	N1—N2—C5—N3	1.1 (14)
N2^i^—Ag1—N1—N2	−2.1 (11)	Ag1^i^—N2—C5—N3	−171.8 (9)
O2—Ag1—N1—N2	120.4 (8)	N1—N2—C5—C6	179.2 (11)
O1—Ag1—N1—N2	169.4 (7)	Ag1^i^—N2—C5—C6	6(2)
C4—N1—N2—C5	−1.3 (13)	C4—N3—C5—N2	−0.4 (14)
Ag1—N1—N2—C5	−172.7 (8)	C4—N3—C5—C6	−178.5 (11)
C4—N1—N2—Ag1^i^	173.2 (7)	N2—C5—C6—C7'	74 (3)
Ag1—N1—N2—Ag1^i^	1.9 (10)	N3—C5—C6—C7'	−108 (2)
Ag1—O2—N4—O1	−5.8 (14)	N2—C5—C6—C8'	−88 (3)
Ag1—O2—N4—O3	−179.6 (12)	N3—C5—C6—C8'	90 (3)
Ag1—O1—N4—O2	5.8 (14)	N2—C5—C6—C7	104 (2)
Ag1—O1—N4—O3	179.7 (12)	N3—C5—C6—C7	−78 (2)
N2—N1—C4—N3	1.0 (12)	N2—C5—C6—C8	−128.8 (19)
Ag1—N1—C4—N3	170.1 (8)	N3—C5—C6—C8	49 (2)

Symmetry codes: (i) −*x*+1, −*y*+1, −*z*+1.

### Hydrogen-bond geometry (Å, °)

**Table:**

*D*—H···*A*	*D*—H	H···*A*	*D*···*A*	*D*—H···*A*
N3—H3···O1^ii^	0.89	2.06	2.93 (1)	167

Symmetry codes: (ii) −*x*+3/2, *y*−1/2, −*z*+1/2.
